# Low-level laser therapy in patients with Burning Mouth Syndrome: A double-blind, randomized, controlled clinical trial

**DOI:** 10.4317/jced.55517

**Published:** 2019-02-01

**Authors:** Juliana-Cassol Spanemberg, Juan-Jose Segura-Egea, Eugenia Rodríguez-de Rivera-Campillo, Enric Jané-Salas, Fernanda-Gonçalves Salum, Jose López-López

**Affiliations:** 1 PhD. Postdoctoral Research Fellow. Specialist in Stomatology and Public Health. Department of Odontoestomatology - Faculty of Medicine and Health Sciences (School of Dentistry); 2 PhD, MD, DDS, Doctor Specialist in Stomatology. Department of Stomatology, School of Dentistry, University of Seville, Spain; 3 MD, DDS, PhD, Dermatologist and Dentist. Professor of Oral Pathology, School of Dentistry, University of Barcelona, Spain; 4 MD, DDS, PhD. Doctor, Specialist in Stomatology. Professor of Oral Pathology, School of Dentistry, University of Barcelona, Spain / Oral Health and Masticatory System Group (Bellvitge Biomedical Research Institute) IDIBELL, University of Barcelona, Spain; 5 PhD, Senior Lecturer, School of Dentistry, Oral Medicine Division, São Lucas Hospital, Pontifical Catholic University of Rio Grande do Sul (PUCRS), Porto Alegre, RS, Brazil; 6 Facultative Director and Clinical Head of the Surgical Medical Area of the Odontological Hospital University of Barcelona

## Abstract

**Background:**

Evaluate the effect of LLLT in the treatment of burning mouth syndrome (BMS).

**Material and Methods:**

Twenty-one BMS patients were randomly assigned to two groups: 12 in the laser group (LG) and 9 in the control group (CG). Patients in the LG underwent 2-week sessions of LLLT for 4 weeks. The spot tip area of this tool is 0.088cm2, semi-conductor GaAlAs, with a wavelength of 808nm ±5nm (infrared), 200 mW output power, 1.97W/cm2 of power density, 3 J energy per point and application time 15 seconds per point. LLLT was applied punctually, in continuous emissions, on each of the sites where there was a symptom. Symptoms were evaluated with a visual analogue scale (VAS) and patient psychological profiles were assessed using the Hospital Anxiety-Depression Scale. No side effects were recorded. Statistical analysis was carried out via ANOVA and logistic regression analysis.

**Results:**

The initial VAS score mean was 8.9 for the LG and 8.3 for the CG (*p* >0.05). After the eighth session the VAS score was 5.5 and 5.8 respectively, and at two months it was 4.7 and 5.1 respectively. Improvement variables were established by dichotomizing the pain scales. We obtained levels of significance for the improvement variable for the LG at the two-month follow-up (*p*=0.0038) and for the univariate analysis of the treatment. The improvement was marginally significant in the multivariant analysis of: dry mouth, dysgeusia, pain and the treatment (*p*=0.0538).

**Conclusions:**

LLLT may be an alternative treatment for the relief of oral burning in patients with BMS.

** Key words:**Burning mouth syndrome, oral pain, laser dentistry, laser therapy, low intensity laser therapy.

## Introduction

Burning mouth syndrome (BMS) is a common pathology. It is characterized by a stinging sensation or sometimes even pain, both of which in the absence of associated pathology or lesions (1). Those who suffer from BMS have symptoms of varying degrees and the intensity can be attributed to their general clinical status, especially with respect to psychological factors (2-4), which may also be linked to the sleep disorders of some patients (5). The lack of unified criteria makes the diagnosis of BMS difficult and causes variation in epidemiological data, depending on the researcher who is analyzing such data (1,6,7). Almost all of the literature mentions a clear predominance in women, and the average age of those affected is between 50 and 60 years old, although it can appear at younger ages (7-9). BMS is clinically manifested with a stinging, burning, and painful sensation that is mostly continuous throughout the day. This sensation is chronic and it is observed at different localizations within the oral cavity, without the presence of any lesion that could justify such symptoms, or any clinical or histological changes (1,6,9). Patients describe a feeling of dry mouth and taste alterations, such as a bitter or metallic taste (10). The description of the symptoms varies for each patient, although the majority of patients describe it as chronic and unbearable (9-11). Food that is spicy or very hot, drinks, stress and fatigue are all factors that are most frequently reported by patients to the worsen the symptoms (1,2). The discomfort tends to be continuous or intermittent and may increase throughout the day (9-11).

BMS has a multifactorial origin. Systemic factors (9,12), local factors (9,10,13-15) and psychological factors such as stress, anxiety and depression (3,5,13,14,16-18) are all possible causes. However on rare occasions BMS can be directly linked to an etiopathogenic factor that acts either locally or systemically (1). The treatment of BMS continues to pose serious problems; it is still not clear which etiopathogenic factors are responsible for such pathology, thus making it difficult to make therapeutic advances. The main objective of treatment is to control the multiples factors that are related to BMS, therefore decreasing the symptoms that patients report (11).

The therapeutic properties of laser radiation have been studied for many years, especially with respect to certain indications and contraindications. Non-ablative laser therapy, also known as clinical laser, or low-level laser therapy (LLLT) was developed in 1965 by Sinclair and Knoll. Because of the energy density and the wavelength, low-level lasers are used in medicine, due to their bio-modulating action and their ability to penetrate tissue (19). Different studies have demonstrated the analgesic, anti-inflammatory and repairing effect that this type of radiation has on tissues, but there is a need for more research to strengthen the data from these trials and establish the most efficient clinical protocols (20-23).

To date it has been verified that low-level laser radiation therapy can be effective in decreasing the symptoms of patients with BMS (20,21,24-26). Santos *et al.* (2011) (24), treated 10 patients with BMS with weekly sessions of LLLT for a period of 10 weeks, using the InGaAIP laser diode in continuous mode. They used a wavelength of 660 nm, 40 mW, 20 J/cm2 of dosimetry and 0.8 J per point for 10 seconds. The intensity of the symptoms was assessed at all of the sessions with a visual analog scale (VAS). The patients reported improvement after the laser treatment, with a reduction in symptoms of up to 50% at the tenth session period. The analgesic effect of the laser radiation is due to the inhibition of nociceptive mediators and the release of endogenous analgesic substances, such as endorphins, by the Central Nervous System (CNS), which works to inhibit the transmission of painful stimuli (27,28). As an immediate effect, after the application of the laser an elevation in the cell membrane potential occurs, as well as the reduction of the speed of nerve impulse conduction (28).

On the other hand, it is important to stress that low power laser therapy is a non-invasive treatment; it is well tolerated by patients and effective for acute and chronic pain. If we start from the premise that patients with BMS should be treated to improve their quality of life, even if this implies that they are not completely cured, and when considering that the objective of treatment is to improve symptoms, given that they are difficult to completely control, LLLT can be a possible alternative with no side effects.

Based on what has been previously mentioned, the hypothesis is that the low-power laser can be effective in controlling the symptoms of the BMS and that its contraindications are minimal. In order to test such hypothesis we pose the objective of clinically evaluating the effect of low-level laser radiation on the reduction of symptoms in patients with BMS, as well as evaluating the degree of anxiety of patients before starting the treatment and at the two-month follow-up.

## Material and Methods

This is a clinical trial (prospective, randomized, double-blind, placebo-controlled). The present study has been approved by the Research Ethics Committee at the DHUB and by local committees, based on the Declaration of Helsinki. Each of the participants in the study signed an informed consent form. The sample comprised 21 patients of both sexes with BMS diagnosis. The study population consisted of patients who were treated in the postgraduate program of Medicine, Surgery and Implantology and Dentistry in Oncology and Immunocompromised Patients at the School of Dental Medicine of the University of Barcelona. Their clinical activity was recorded at the Dental Hospital - University of Barcelona (DHUB).

The study included patients over 40 years old who reported symptoms of burning or pain in the oral mucosa of at least 3 months of duration. Patients with uncontrolled systemic diseases (ASA III, IV) or without clinical activity of BMS, or a VAS score below 3 out of 10. Those patients who did not agree to participate in the study were also excluded. All the patients received instructions regarding oral hygiene, mucosal hydration and were advised to avoid spicy and citric foods, as well as alcoholic beverages and tobacco.

-Trial Procedures

A general medical history was obtained for all patients, as well as a specific medical history with respect to BMS (evolution time, level of pain, treatment, etc.). The Hospital Anxiety and Depression Scale (HADS) were also used at the beginning of the treatment and at the two-month follow-up. A questionnaire was used to assess the discomfort associated with the laser technique ( Table 1). Two groups were randomly created: Laser Group (LG): n= 12; Control Group (CG): n=9.

**Table 1:**
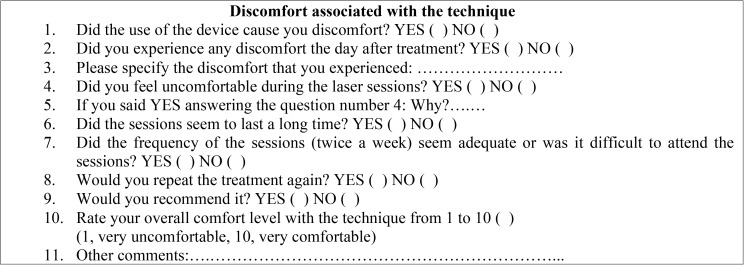
Questionnaire used for patients with BMS to obtain information about the level of discomfort associated with the laser technique.

**Figure 1:**
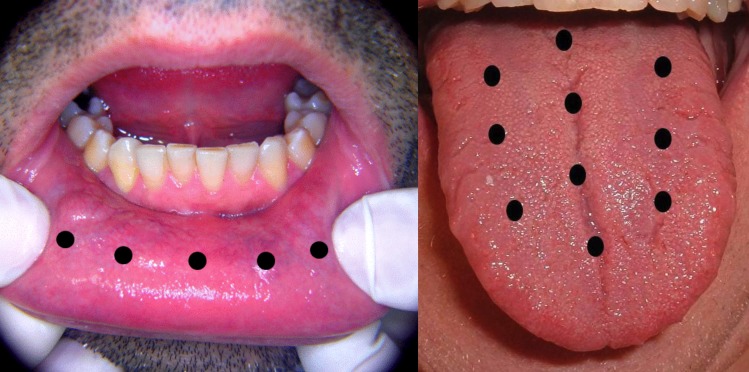
Diagram of points to illustrate the application of the low-level laser on the lower labial mucosa membrane and on the dorsal surface of tongue.

A bi-weekly application of a low-level laser diode (Thor Laser®) was performed on the LG for 4 weeks; total of 8 sessions. The spot tip area of this tool is 0.088cm2, semi-conductor GaAlAs, with a wavelength of 808nm ±5nm (infrared), 200 mW output power, 1.97W/cm2 of power density, 3 J energy per point and application time 15 seconds per point. LLLT was applied punctually, in continuous emissions, on each of the sites where there was a symptom. The number of laser application points was determined by the areas mentioned by the patients (Fig. 1): tip of the tongue: 3 points; lateral border of the tongue: 4 points; dorsal surface of the tongue: 10 points; buccal mucosa: 8 points; labial mucosa: 5 points, hard palate: 8 points, soft palate: 3 points; gingiva or alveolar mucosa: 3 points by sextant. The patients were reevaluated two months after the end of the treatment.

We used the same protocol for the control group as we did for the experimental group (time and application values) but the laser was deactivated for the entirety of the consultation, checked by means of a power meter prior to the applications. Neither the patient nor the researcher knew if the laser was activated or not.

-Statistical Analysis

The variable responses used in the present study, VAS and the percentage of symptom improvement, were summarized as a mean and standard deviation. All data were analyzed with the Shapiro-Wilk W-Test to assess the normality. Since the data did not have normal distributions, the Friedman ANOVA followed by the Wilcoxon Matched Pairs Test were applied to analyze the difference between the groups. Significance was accepted at *p*<0.05 for all tests. The software used was SPSS (Stat Software, Inc., Tulsa, OK).

## Results

All the patients in the sample (n=21) completed the study (12 in the LG and 9 in the CG), there were 20 women (95%) and one male, who participated in the control group. The average age was 66.3 (range of 61-81, SD=6.9), showing no differences between the two groups (LG=66.3±7.52 and CG=66.4±6.31; *p*=6.699). The rest of the clinical variables relating to the symptoms of burning did not show differences between the two groups ( Table 2). There was only marginal significance with regard to the burning sensation (*p*= 0.0653) ( Table 2). With respect to the evolution time of the symptoms, there were no differences between the two groups; the average was 59 months for the LG and 56 months for the CG, with a maximum range of 10.8 years and 8 months.

**Table 2:**
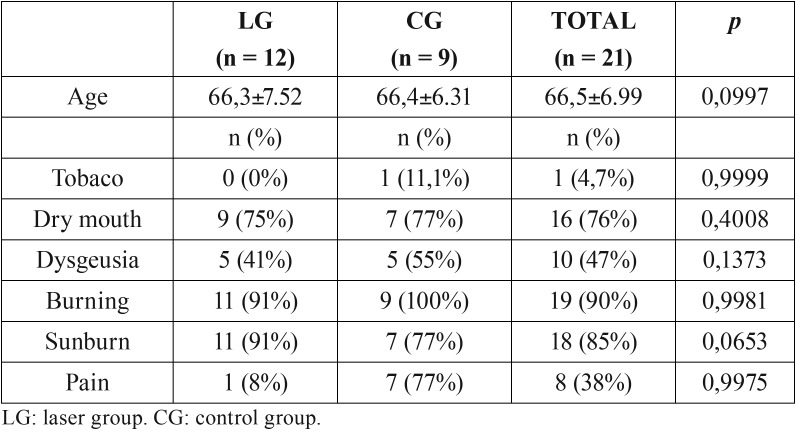
Descriptive variables related to BMS symptoms.

**Figure 2:**
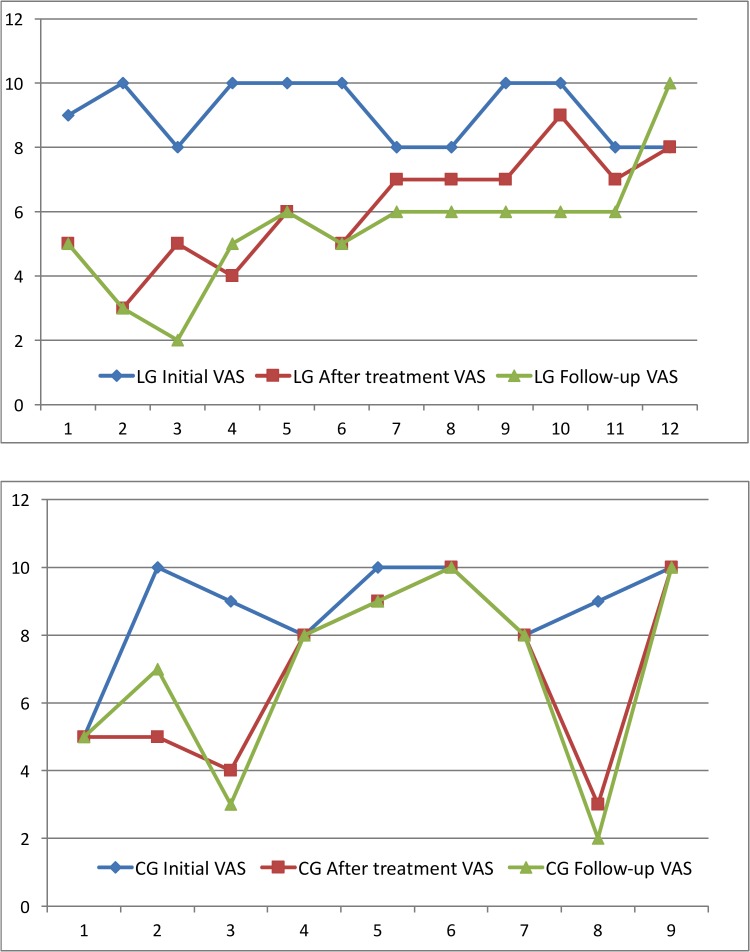
Dates of basal VAS, after treatment (8 applications) and follow-up (two months). LG: Laser group; CG: Control group.

The average value of the initial VAS was 8.9 for the LG and 8.3 for the CG; at the eighth session it was 5.5 and 5.8 respectively and at the two-month follow-up it was 4.7 and 5.1 respectively (Fig. 2). All patients were taking at least one medication: one patient took four different medications, four patients took three different medications, and eight patients took two different medications. It is worth noting that 6 patients (28%) were taking psychotropic medication. With regard to the medical history, 2 patients had no other relevant medical history, but 8 patients (38%) suffered from three or more concomitant diseases, 6 of them were cardiovascular diseases, 7 had high blood pressure and 8 of them were taking antiplatelet drugs. Ten of the patients (47%) had a completely normal oral examination. Six showed signs of white tongue; one had white tongue, geographic tongue and actinic cheilitis, this patient corresponded to the LG, and went from a VAS score of 10 to a VAS score of 3 over the two-month period.

In reference to the clinical manifestations, 8 patients were labeled with Lamey Type I BMS (14), 13 were described as having Type II, and none of the patients met criteria for Type III. There were no differences between the control and treatment groups. With respect to the localization of the symptoms, 20 of the 21 patients were affected on the tongue at various localizations: 13 on the tip of the tongue, the dorsal surface and the lateral borders, 13 on the tip of the tongue and the dorsal surface, 2 on the tip of the tongue and the lateral borders, 18 on just the tip of the tongue, 1 on just the dorsal surface and 1 on just the lateral borders. The palate was affected in 9 patients (43%) and 8 of them coincided with some kind of manifestation on the tongue. The lips were affected in 11 patients (52%), but in all cases there was some kind of concomitant manifestation on the tongue. A total of 6 patients had manifestations on areas besides the tongue, lip or palate; but in all cases there were concomitant manifestations on the tongue, especially on the tip of the tongue. There were no differences between the groups and their response, based on the localization of the symptoms. All the patients, without any exceptions, showed a good level of tolerance to the technique utilized and 19 of them (90%) would repeat treatment again.

In order to dichotomize the pain scales, we established improvement variables for each one of the measurements; improvement was considered to have taken place when the score decreased 2 points or more as compared to the previous measurement. We likewise established the improvement variable of 30% at the two-month follow-up, if the initial VAS score decreased 30%. There was a 50% improvement, if the initial value decreased 50%, and finally the VAS≤5 variable, if this was the result obtained at the two-month follow-up visit. Based on these variables we obtained clear significance for the LG in the improvement variable after the two month period, showing *p*=0.0038 in the univariate analysis related to the implemented treatment ( Table 3), and proving to be marginally significant in the multivariate analysis carried out with respect to: dry mouth, dysgeusia, pain and the implemented treatment (*p*=0.0538) ( Table 4).

**Table 3:**
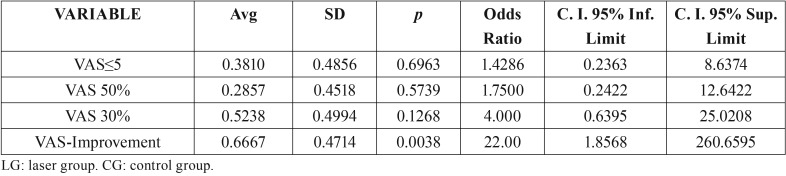
Descriptive analysis of the variables at the two-month mark: VAS≤5, VAS 50%, VAS 30%, VAS-Improvement. (0 = CG, without laser, 1 = LG, with LLLT).

**Table 4:**
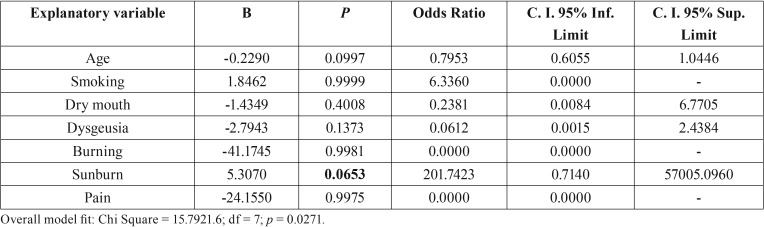
Multivariate logistic regression analysis of the influence of the independent variables age, smoking (0 = non-smoker, 1 = smoker), dry mouth (0 = absent, 1 = present), dysgeusia (0 = absent, 1 = present), burning (0 = absent, 1 = present), on the dependent variable “treatment group” (0 = CG, without laser, 1 = LG, with LLLT).

In regard to the psychological profiles of the patients, there were no statistically significant differences between the groups before or after the treatment ( Table 5).

**Table 5:**
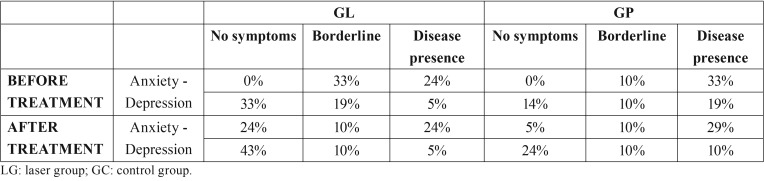
Percentage of patients according to scores on the Hospital Anxiety and Depression Scale (HADS). There were no differences between the groups before or after the treatment.

## Discussion

The present study has clinically assessed LLLT effects in the treatment of patients with burning mouth syndrome. The patients in this study were treated with LLLT after having tried treatments with other types of medication and not having any satisfactory relief of the symptoms with such treatment.

In the series with 10 patients and no control group, published by Santos *et al.* (2011) (24), the use of the low-level laser showed a reduction in the average percentage of the intensity of the pain of up to 58.2% according to VAS scale values at the tenth session. The initial scores from the visual analog scale were significantly lower after the forth laser application, when compared to those values from the first session. Kato *et al.* (2010) (25) utilized the LLLT on the localization where the patients reported symptoms for 11 patients with BMS. The affected areas were irradiated once a week, in continuous mode, with a wavelength of 790 nm and 6 J/cm2 of dosimetry. The intensity of the symptoms was recorded by means of the VAS in each one of the three sessions, as well as six weeks after the treatment had ended. At the end of the study the authors verified that the experimental group showed significant symptom improvement as compared to their status at the beginning of the treatment. The patients reported a decrease of 80.4% in the intensity of the symptoms after the laser radiation treatment, which suggests that LLLT can be an alternative for BMS.

Spanemberg *et al.* (2015) (26) aimed to clinically assess the effect of different LLLT protocols in the treatment of BMS patients and presented three protocols, red and infrared, in a randomized, blind, placebo-controlled study, seeking positive outcomes for the management of BMS. A diode laser was used in 78 BMS patients punctually on each of the sites with the symptom. Patients were randomly assigned into four groups: Infrared weekly (830 nm, 100 mW, 5 J, 176  J/cm2 176  J/cm2, 50 s, LLLT weekly sessions, 10 sessions); Infrared three times a week (830 nm, 100 mW, 5 J, 176  J/cm2 176  J/cm2, 50 s, three LLLT weekly sessions, 9 sessions); Red laser (685 nm, 35 mW, 2 J, 72  J/cm2 72  J/cm2, 58 s, three LLLT weekly sessions, 9 sessions); and control-group. There was significant reduction of the symptoms in all groups at the end of the treatment (*P*<0.001), which was maintained in the follow-up (*P*<.0001). The results shows that the protocols used infrared laser were very effective in reducing BMS symptoms.

However, authors like Vukoja *et al.* (27) were incapable of confirming such results and they suggest that the therapeutic benefit of the laser in patients with BMS is caused by the placebo effect, and its duration is limited over time. Many BMS patients mention decrease in symptoms and psychological improvement due to the fact that they have been receiving medical attention and advice. Throughout the literature we find few placebo-controlled clinical trials that utilize the low-level laser in patients with BMS. The results were satisfactory in the study that we have presented, and the fact that it was a double-blind study allows us to minimize the interference of the placebo in the results as much as possible.

Studies have shown that BMS can have neuropathic origin (10,12,16). López-Jornet *et al.* (10) suggest that hyperactivity of trigeminal nociceptive pathways can produce an intense response to the action of irritating factors, leading to the occurrence of BMS symptoms. The relief of symptoms of a burning sensation provided by the laser therapy is probably caused by nociceptive modulation due to the liberation of endorphins and enkephalins (28-31). In addition to this, the laser diode can reduce inflammation by means of increasing the production of PG-I2 (31) and PG-E2 (32), thus increasing the formation of blood vessels (31). In this study, the majority of the patients experienced burning on the tongue, in addition to other localizations, and although the LLLT was topically administered, the laser is also capable of increasing the blood flow when it is applied directly to the affected areas (33).

López-Jornet *et al.* (34) investigated the quality of life of 216 patients with oral mucosa diseases. The lowest scores were found in patients with burning mouth syndrome. Ni Riordain *et al.* (35) observed that after treatment, patients with BMS exhibited improvement in quality of life, thus demonstrating that the disorder has a negative impact on physical, mental and social well-being. Souza *et al.* (36) described the impact of BMS on health-related quality of life in patients with this disease. When evaluating the psychological profile of the patients, although we did not find statistically significant differences, the HADS scores showed that anxiety decreased after the low-level laser treatment. A possible explanation for this could be the decrease, although slight in some cases, in the symptoms of burning and pain experienced by patients before beginning the laser treatment. Femiano *et al.* in 2004 (18) stated that the increase in BMS pain is associated with the frustration of being affected by the disease and/or the patients’ dependence on others when they are faced with problems that are not resolved. This thus supports the evidence that psychological stress can be a significant factor in the development of BMS, at least in some cases.

Different researchers have evaluated the possible adverse effects of LLLT, but no significant results were found (26,28,37). Our study coincides with these data. All of the patients had a satisfactory response to the treatment. There were no relevant side effects and the majority of patients completed the treatment without interruption. Only one patient abandoned the study due to discomfort experienced with the technique, this patient was in the laser group. All of the other individuals showed a good level of tolerance throughout the study and would repeat the treatment.

The parameters used in our study aimed at the analgesic effect, once neuropathic factors have been suggested as the cause of BMS. Due to the variability of options regarding LLLT parameters, we believe that several protocols, besides the one applied in the present study, could bring beneficial results to the BMS patients.

Based on the results of this study we can conclude that for a small number of patients with BMS the results of the low-level laser treatment were satisfactory. This treatment could be an acceptable alternative to psychoactive drugs. We believe that studies with a long follow-up period, as well as those with a large number of participants are fundamental to confirm the effectiveness of this therapeutic alternative.

## References

[1] Zakrzewska JM, Forssell H, Glenny AM (2003). Interventions for the treatment of burning mouth syndrome: a systematic review. J Orofac Pain.

[2] Forssell H, Teerijoki-Oksa T, Kotiranta U, Kantola R, Bäck M, Vuorjoki-Ranta TR (2012). Pain and pain behavior in burning mouth syndrome: a pain diary study. J Orofac Pain.

[3] Abetz LM, Savage NW (2009). Burning mouth syndrome and psychological disorders. Aust Dent J.

[4] Ritchie A, Kramer JM (2018). Recent Advances in the Etiology and Treatment of Burning Mouth Syndrome. J Dent Res.

[5] Adamo D, Schiavone V, Aria M, Leuci S, Ruoppo E, Dell'Aversana G (2013). Sleep disturbance in patients with burning mouth syndrome: a case-control study. J Orofac Pain.

[6] Evans RW, Drage LA (2005). Burning mouth syndrome. Headache.

[7] Danhauer SC, Miller CS, Rhodus NL, Carlson CR (2002). Impact of criteria-based diagnosis of burning mouth syndrome on treatment outcome. J Orofac Pain.

[8] Tarkkila L, Linna M, Tiitinen A, Lindqvist C, Meurman JH (2001). Oral symptoms at menopause-the role of hormone replacement therapy. Oral Surg Oral Med Oral Pathol Oral Radiol Endod.

[9] Rodríguez-de Rivera-Campillo E, López-López J (2013). Evaluation of the response to treatment and clinical evolution in patients with burning mouth syndrome. Med Oral Patol Oral Cir Bucal.

[10] López-Jornet P, Camacho-Alonso F, Andujar-Mateos P, Sánchez-Siles M, Gómez-Garcia F (2010). Burning mouth syndrome: an update. Med Oral Patol Oral Cir Bucal.

[11] Spanemberg JC, Cherubini K, de Figueiredo MA, Yurgel LS, Salum FG (2012). Aetiology and therapeutics of burning mouth syndrome: an update. Gerodontology.

[12] Koszewicz M, Mendak M, Konopka T, Koziorowska-Gawron E, Budrewicz S (2012). The characteristics of autonomic nervous system disorders in burning mouth syndrome and Parkinson disease. J Orofac Pain.

[13] Lamey PJ, Lewis MAO (1989). Oral medicine in practice: Burning mouth syndrome. R Dent J.

[14] Lamey PJ (1996). Burning mouth syndrome. Dermatolo Clin.

[15] Klasser GD, Fischer DJ, Epstein JB (2008). Burning mouth syndrome: recognition, understanding, and management. Oral Maxillofac Surg Clin North Am.

[16] Lauria G, Majorana A, Borgna M, Lombardi R, Penza P, Padovani A (2005). Trigeminal small-fiber sensory neuropathy causes burning mouth syndrome. Pain.

[17] Mock D, Chugh D (2010). Burning mouth syndrome. Int J Oral Sci.

[18] Femiano F, Gombos F, Scully C (2004). Burning Mouth Syndrome: open trial of psychotherapy alone, medication with alpha-lipoic acid (thioctic acid), and combination therapy. Med Oral.

[19] Henriques AC, Maia AM, Cimões R, Castro JF (2008). The laser therapy in Dentistry: properties, indications and current aspects. Odontologia Clín Científ.

[20] Al-Maweri SA, Javed F, Kalakonda B, AlAizari NA, Al-Soneidar W, Al-Akwa A (2017). Efficacy of low-level laser therapy in the treatment of burning mouth syndrome: A systematic review. Photodiagnosis Photodyn Ther.

[21] Spanemberg JC, Figueiredo MA, Cherubini K, Salum FG (2016). Low-level Laser Therapy: A Review of Its Applications in the Management of Oral Mucosal Disorders. Altern Ther Health Med.

[22] Abramoff MM, Lopes NN, Lopes LA, Dib LL, Guilherme A, Caran EM (2008). Low-level laser therapy in the prevention and treatment of chemotherapy-induced oral mucositis in young patients. Photomed Laser Surg.

[23] Kuhn A, Porto FA, Miraglia P, Brunetto AL (2009). Low-level infrared laser therapy in chemotherapy-induced oral mucositis: a randomized placebo-controlled trial in children. J Pediatr Hematol Oncol.

[24] Santos LF, Carvalho AA, Leão JC, Cruz Perez, Castro JF (2011). Effect of low-level laser therapy in the treatment of burning mouth syndrome: a case series. Photomed Laser Surg.

[25] Kato IT, Pellegrini VD, Prates RA, Ribeiro MS, Wetter NU, Sugaya NN (2010). Low-level laser therapy in burning mouth syndrome patients: a pilot study. Photomed Laser Surg.

[26] Spanemberg JC, López JL, de Figueiredo MA, Cherubini K, Salum FG (2015). Efficacy of low-level laser therapy for the treatment of burning mouth syndrome: a randomized, controlled trial. J Biomed Opt.

[27] Vukoja D, Alajbeg I, Vučićević Boras V, Brailo V, Alajbeg IZ, Andabak Rogulj A (2011). Is effect of low-level laser therapy in patients with burning mouth syndrome result of a placebo?. Photomed Laser Surg.

[28] Moore K (2004). Lasers and pain treatment. Laser Part Clinix.

[29] Pozza DH, Neto NR, Sobrinho JBM, Oliveira MG, Marzola C (2009). Analgesic effect analysis of the laser therapy red laser (660nm) and infrared laser irradiation (830nm) in healthy tissues of the mice. Rev Acad Tirad Odontol.

[30] Hagiwara S, Iwasaka H, Okuda K, Noguchi T (2007). GaAlAs (830 nm) low-laser enhances peripheral endogenous opioid analgesia in rats. Lasers Surg Med.

[31] Tam G (1999). Low power laser therapy and analgesic action. J Clin Laser Med Surg.

[32] Shimizu N, Yamaguchi M, Goseki T, Shibata Y, Takiguchi H, Iwasawa T (1995). Inhibition of prostaglandin E2 and interleukin 1-beta production by low-power laser irradiation in stretched human periodontal ligament cells. J. Dent. Res.

[33] Bossini PS, Fangel R, Habenschus RM, Renno AC, Benze B, Zuanon JA (2009). Low-level laser therapy (670 nm) on viability of random skin flap in rats. Lasers Med. Sci.

[34] López-Jornet P, Camacho-Alonso F, Lucero-Berdugo M (2009). Measuring the impact of oral mucosa disease on quality of life. Eur J Dermatol.

[35] Ni-Riordain R, Moloney E, O'Sullivan K, McCreary C (2010). Burning mouth syndrome and oral health-related quality of life: is there a change over time?. Oral Dis.

[36] Souza FTA, Santos TPM, Bernardes VF, Teixeira AL, Kümmer AM, Silva TA (2011). The impact of burning mouth syndrome on health-related quality of life. Health Qual Life Outcomes.

[37] Bjordal JM, Bensadoun RJ, Tunèr J, Frigo L, Gjerde K, Lopes-Martins RA (2011). A systematic review with meta-analysis of the effect of low-level laser therapy (LLLT) in cancer therapy-induced oral mucositis. Support Care Cancer.

